# Inactivation Rates for Airborne Human Coronavirus by Low Doses of 222 nm Far-UVC Radiation

**DOI:** 10.3390/v14040684

**Published:** 2022-03-25

**Authors:** David Welch, Manuela Buonanno, Andrew G. Buchan, Liang Yang, Kirk D. Atkinson, Igor Shuryak, David J. Brenner

**Affiliations:** 1 Center for Radiological Research, Columbia University Irving Medical Center, New York, NY 10032, USA; mb3591@cumc.columbia.edu (M.B.); is144@cumc.columbia.edu (I.S.); djb3@cumc.columbia.edu (D.J.B.); 2 School of Engineering and Materials Science, Queen Mary University of London, London E1 4NS, UK; a.buchan@qmul.ac.uk; 3 School of Water, Energy and Environment (SWEE), Cranfield University, Bedford MK43 0AL, UK; liang.yang@cranfield.ac.uk; 4 Faculty of Energy Systems and Nuclear Science, Ontario Tech University, Oshawa, ON L1G 0C5, Canada; kirk.atkinson@uoit.ca

**Keywords:** ultraviolet radiation, far-UVC, coronavirus, airborne, radiation transport, computational fluid dynamics

## Abstract

Recent research using UV radiation with wavelengths in the 200–235 nm range, often referred to as far-UVC, suggests that the minimal health hazard associated with these wavelengths will allow direct use of far-UVC radiation within occupied indoor spaces to provide continuous disinfection. Earlier experimental studies estimated the susceptibility of airborne human coronavirus OC43 exposed to 222-nm radiation based on fitting an exponential dose–response curve to the data. The current study extends the results to a wider range of doses of 222 nm far-UVC radiation and uses a computational model coupling radiation transport and computational fluid dynamics to improve dosimetry estimates. The new results suggest that the inactivation of human coronavirus OC43 within our exposure system is better described using a bi-exponential dose–response relation, and the estimated susceptibility constant at low doses—the relevant parameter for realistic low dose rate exposures—was 12.4 ± 0.4 cm^2^/mJ, which described the behavior of 99.7% ± 0.05% of the virus population. This new estimate is more than double the earlier susceptibility constant estimates that were based on a single-exponential dose response. These new results offer further evidence as to the efficacy of far-UVC to inactivate airborne pathogens.

## 1. Introduction

Diseases transmitted through airborne routes have been a public health issue long before the current COVID-19 pandemic. One of the most prominent and deadly airborne diseases worldwide is tuberculosis, which was demonstrated to spread via airborne droplets in the 1950s [1]. Other diseases transmitted through airborne routes include measles, smallpox, influenza [2], and the common cold [3]. Current evidence also points towards the airborne route for COVID-19 transmission [4]. Undoubtedly, mitigating the risk of airborne disease transmission is crucial to both current and future public health goals.

Vaccination is an effective means of preventing infection from many diseases capable of spreading through airborne routes; however, vaccine development, testing, production, and distribution requires significant time and capital investment [5]. Ultraviolet radiation is an established and viable strategy that can complement vaccination and other engineering approaches to prevent airborne disease transmission [6]. Because of its non-targeted mode of action, UV is effective against most airborne pathogens and is likely to be effective against newly emerging viruses [7]. Crucially, UV differs from the more commonly considered approaches to prevent airborne transmission—lockdowns, mask wearing, and vaccination—in that it does not require active decisions on the part of the general public.

Currently, the primary application of ultraviolet radiation for air disinfection is through upper room ultraviolet germicidal irradiation (UVGI) systems [8]. UVGI systems typically use lamps emitting primarily at 254 nm. Because direct exposure to 254 nm radiation can potentially cause harm to the eyes and skin, UVGI systems restrict radiation to the upper portion of the room and are carefully installed to minimize emissions into human-occupied spaces [9].

A new concept in the use of ultraviolet radiation for airborne disinfection utilizes the wavelength range from 200–235 nm, often referred to as far-UVC [10,11,12,13,14,15]. Research has demonstrated these wavelengths are at least as effective for microbial inactivation as conventional 254 nm UV radiation [10,11,14,15,16,17,18,19,20,21], yet potentially without the associated health hazards [10,11,12,13,22,23,24,25,26,27,28,29,30,31,32,33]. Thus, far-UVC can potentially be directly used in occupied indoor locations to continuously disinfect the air and exposed surfaces [14,15]. Recent modeling work has predicted that application of far-UVC within a room can offer an individual protection against airborne viruses similar to that provided by wearing an N95 mask [34].

Our earlier laboratory work demonstrated the efficacy of inactivation of aerosolized viruses with 222 nm far-UVC, first for airborne influenza A virus H1N1 [15] and, more recently, for airborne human coronaviruses 229E and OC43 [14]. In the current work, we (a) studied a much wider range of far-UVC doses and (b) used a computational model to generate improved dosimetry estimates. The wider dose range is of particular importance in that in real-life scenarios the far-UVC exposures will be delivered at very low dose rates—effectively repeating multiple times the low-dose part of the dose–response relation [35]—and thus the overall effectiveness will be determined by the low-dose component of the dose–response relation.

## 2. Materials and Methods

### 2.1. Viral Strain

All experiments were performed using the human coronavirus OC43 (HCoV-OC43) (ATCC VR-1558). The HCoV-OC43 virus appears to be a suitable surrogate for SARS-CoV-2, with comparable physical and genomic size [36], and has been previously used by our group [14] and others [37] for ultraviolet radiation efficacy studies. Recent research on SARS-CoV-2 susceptibility with 222 nm radiation on surfaces [20,21] as well as other studies investigating coronavirus susceptibility [38] provide further evidence of similar efficacy between coronaviruses. Details of preparation of this virus and its propagation in host WI-38 normal lung cells (ATCC CCL-75) are available in the manuscript by Buonanno et al. (2020) [14].

### 2.2. Benchtop Aerosol Irradiation Chamber

Virus inactivation experiments were performed using our custom-built benchtop aerosol irradiation chamber. The layout and operation of this system was previously described in detail [14,15]. This one-pass exposure system integrates the generation, exposure, and collection of aerosols containing viruses within a single chamber. The benchtop system includes a nebulizer for aerosol generation, dry and humidified air inputs to maintain humidity, particle size monitoring, an exposure volume (279 mm tall × 254 mm wide × 63 mm deep) with a UV transmitting window to enable UV exposure within the chamber, and a vacuum pump to move the aerosol through the system. Some modifications have been made to the system since our previous report [14] in order to enable a broader range of exposure doses and improve overall system operation. First, the chamber previously used a UV transmitting plastic as the exposure window on the chamber. For the tests described here, the chamber window is 6 mm of quartz glass (GE Type 124, Technical Glass Products, Painesville, OH, USA). The measured transmission of the 6 mm quartz window for 222 nm radiation was 72%. Second, the rear reflector used within the chamber is now Anolux UVS aluminum (Anomet, Brampton, ON, Canada). Anolux has a specular reflectance of approximately 60% for 222 nm radiation. Third, aerosol collection was performed using 37 mm gelatin membrane filters (SKC Inc., Eighty Four, PA, USA) held within a plastic air monitoring cassette (37 mm SureSeal Casette, SKC Inc.). Previously, our system utilized a BioSampler (SKC Inc.) for aerosol collection, which also controlled the flow rate through the chamber to be 12.5 LPM. With the change to gelatin filters for collection, it was necessary to incorporate a precision flow orifice (B-47-SS, O’Keefe Controls Co, Monroe, CT, USA) which set the flow rate through the system to 11.6 LPM via choked flow operation conditions using the system vacuum. A vacuum gauge was used to verify choked flow operating conditions for the orifice, and the flow rate through the system was monitored with an inline air flow meter (EW-32461-54, Cole-Parmer, Vernon Hills, IL, USA). Given these system changes, the total time for a particle to traverse the exposure window was approximately 23 s.

### 2.3. Irradiation Chamber Performance

The overall chamber performance was similar to our previous studies testing efficacy of aerosolized virus inactivation [14,15]. The average temperature during testing was 24 °C, and the relative humidity was between 60–70%. The aerosol size distribution, which was measured using an optical particle counter (HAL-HPC601, Hal Technology, Rancho Cucamonga, CA, USA), was again typical of human coughing, breathing, and talking [39], with over 90% of particles less than 1.0 µm diameter.

### 2.4. Far-UVC Lamp and Dosimetry

The far-UVC source used in this study was a 12 W 222-nm KrCl excimer lamp module made by USHIO America (Item #9101711, Cypress, CA, USA). The lamp is equipped with a proprietary optical filtering window to reduce lamp emissions outside of the 222 nm KrCl emission peak. Spectral analysis of the filtered KrCl lamp was performed using a Gigahertz Optik BTS2048-UV light meter (Gigahertz-Optik Inc, Amesbury, MA, USA), and the normalized emission spectrum is provided in Supplemental Figure S1. The lamp was positioned 22 cm away from the exposure chamber window and directed at the center of the window. The intensity of the lamp was measured using a UIT2400 meter (Ushio America Inc., Cypress, CA, USA) equipped with an SED220 detector and W diffuser input optic. The lamp intensity data were input into the radiation transport model to calculate the radiant exposure dose received by particles moving through the system.

### 2.5. Computational Model of Exposure System

A model incorporating radiation transport and computational fluid dynamics (CFD) was used to simulate the exposure dose received by particles representing aerosols containing virus as they traveled through the exposure chamber. The general modeling approach is described in detail elsewhere [34,40]. The model simulated the experimental setup, including the chamber geometry, the position and emission pattern of the far-UVC source, and the flow conditions of the aerosols within the chamber. It resolves the spatially varying flow fields and far-UVC fluence variations within the chamber and includes a particle model for aerosol tracking, enabling the calculation of the far-UVC inactivation of aerosolized human coronavirus in the system. This was achieved by using the WYVERN coupled radiation-CFD code [34], a high-fidelity modeling solver for the Boltzmann transport and Navier–Stokes (with Large Eddy Simulation (LES)) equations.

The simulation tracked 1600 particles, representing virus containing aerosols, through the irradiation chamber. They were initiated at the chamber’s inlet face where they were evenly distributed in a 40 × 40 grid and followed the flow field, as predicted by the model, to the outlet channels. The virus content of a particle was initiated to a unit value, and this decayed according to the strength of the radiation field, at its respective position, within the domain.

Effects from aerosol size have not been included in this model. Given the very short time scales within the chamber, the laminar flows, and small sizes of aerosols, the dominating effect driving their transport is via the air flow velocity. Aerosol transport is therefore modelled based solely on the airflow. Similarly, aerosol dependent radiation transport effects such as shadowing and scattering are not considered in this simulation because of the small size of the aerosols.

### 2.6. Experimental Protocol

As previously described [14], the virus solution in the nebulizer consisted of 1 mL of Modified Eagle’s Medium (MEM, Life Technologies, Grand Island, NY, USA) containing 10^6^–10^7^ 50% Tissue Culture Infection Dose (TCID_50_) of coronavirus, 20 mL of deionized water, and 0.05 mL of Hank’s Balanced Salt Solution with calcium and magnesium (HBSS++). The irradiation chamber was operated with aerosolized virus particles flowing through the chamber and the bypass channel for 5 min prior to each experiment. Sample collection was initiated by changing air flow from the bypass channel to the gelatin filter using the pair of three-way valves. During each sampling time, which lasted for 30 min, the inside of the irradiation chamber was exposed to 222 nm far-UVC light entering through the quartz window.

Variation of the far-UVC dose delivered to aerosol particles was achieved by inserting precision wire meshes between the far-UVC emitting lamp and the exposure chamber. The wire meshes used in this work had open areas of 64% (item number 9656T11), 46% (9656T15), or 31% (9656T18); all meshes were purchased from Mcmaster-Carr (Elmhurst, IL, USA). Each mesh was confirmed to transmit 222 nm far-UVC in agreement with the specified open area percentage. The mesh was placed against the quartz window of the chamber when only a single mesh was required. For exposures using two meshes in series, the second mesh was placed against the output window of the lamp. Zero-dose control studies were conducted with the excimer lamp turned off. Some test conditions required the dose to be altered by reducing the exposure time by 50%; this was done by covering half of the exposure window with a thick card stock which did not transmit UV radiation. Combinations of wire meshes and the 50% reduction in exposure time were utilized to achieve the doses shown in Table 1. After the sampling period was completed, the gelatin filter was dissolved by shaking in 5 mL of PBS pre-warmed at ~30 °C for 5 min, and the solution was used for the virus infectivity assays.

### 2.7. Virus Infectivity Assay

Testing for inactivation followed the same procedure for testing the 50% Tissue Culture Infectious Dose (TCID_50_) assay as described in Buonanno et al. [14,41,42]. Briefly, the collected viral solution was serially diluted (1:10) and overlayed on WI-38 human lung cells seeded the day before the experiment in 96-well plates (10^5^ cells/well); after a two-hour incubation period at 33–34 °C in infectious medium (MEM + 2% heat-inactivated FBS), the infectious medium was aspirated and replaced by fresh medium (MEM + 10% heat-inactivated FBS + pen/strep), and the plate returned to the incubator. Cytopathic effects (CPE) (e.g., vacuolization of cytoplasm and sloughing) were scored 3 or 4 days later, and TCID_50_ was calculated with the Reed–Muench method [41,43].

### 2.8. Data Analysis

The two phase dose–response model used in this work is commonly used in disinfection and microbial inactivation analyses [7]. This model describes a bi-exponential dose–response, where one exponential describes the behavior of a susceptible fraction of the population, and the second exponential describing the response of a more resistant subpopulation:

(1) $$S=\left(1-f\right)e^{-k_{1}D}+fe^{-k_{2}D},$$

where *S* is the non-inactivated (surviving) fraction of the virus, and *D* is the radiant exposure dose in mJ/cm^2^. (1 − *f*) and *f* are respectively the proportions of the sensitive and the resistant subpopulations whose exponential dose responses are respectively defined by parameters *k*_1_ and *k*_2_ (units of cm^2^/mJ). The mono-exponential model was described as

(2) $$S=e^{-kD}.$$

The non-inactivated fraction (*S*) of the virus for each exposure condition was calculated by dividing the TCID_50_ at each dose by the TCID_50_ of the unexposed condition: *S* = TCID_50,UV_/TCID_50,control_. Inactivation values were calculated for each repeat experiment and natural log (ln) transformed to bring the error distribution closer to normal [44]. Robust regression was performed using *R* 4.0.3 software [45] using these normalized ln[*S*] values as the dependent variable and UV dose (D, mJ/cm^2^) as the independent variable. The *nlrob* function in *R* was used to fit the non-linear bi-exponential model (Equation (1)), and the *rlm* function was used to fit the linear single-exponential model (Equation (2)).

The performances of both model versions (Equations (1) and (2)) were compared using the Akaike information criterion with sample size correction (AICc) [46,47], which compares maximized log likelihood values for all models and penalizes model complexity (i.e., extra adjustable parameters). The model with the lowest AICc score is better supported by the data. The “evidence ratio” of each model relative to the other was evaluated using the quantity exp(ΔAICc/2), where ΔAICc is the difference in AICc scores between models.

## 3. Results

### 3.1. Far-UVC Dosimetry

Measurements of the far-UVC lamp angular emission pattern and the intensity change with distance were recorded, and the values were confirmed to be in agreement with the computational radiation transport model for the lamp. Additional details on the validation are available in the previous description of the radiation model [40].

**Figure 1:**
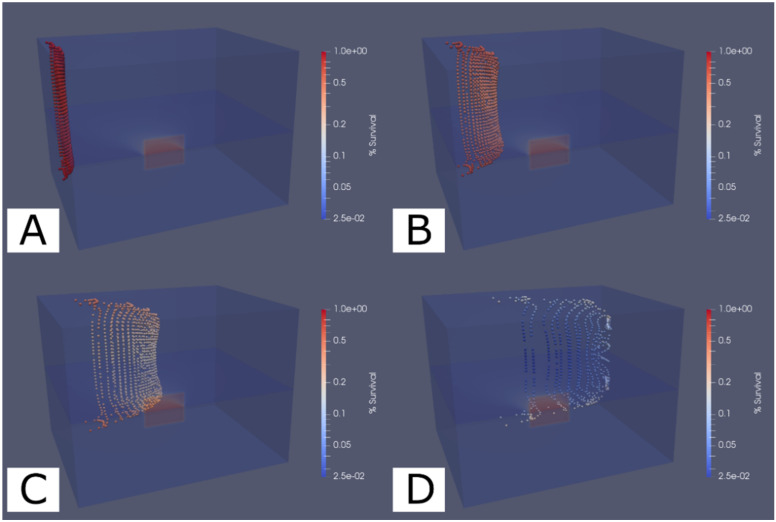
Simulation results of aerosol dosimetry showing the relative survival fraction of virus in aerosols as they traverse across the exposure chamber. Aerosols in the simulation were uniformly distributed across the left side of the exposure volume and released to move through the volume. The aerosol color changing from red to blue indicates an increase in the total radiation flux received by that aerosol over time. Four frames (**A**–**D**) show aerosol position at time instance of 2, 6, 10, and 20 s from entering the chamber. These frames illustrate the flow pattern as well as the virus inactivation for each aerosol. Panel A shows the aerosol particles evenly distributed as they begin into the exposure volume, and panels B and C show the particles progressing across the exposure volume. Panel D shows many of the particles have reached the two chamber outlet ports on the right side, while slower travelling particles are still being exposed. A video of the simulation result is available in the Supplementary Materials.

Selected frames from the dosimetry simulation are shown in Figure 1, and a video of the complete simulation showing motion through the chamber (Video S1) is available with the Supplementary Materials. The images in Figure 1 show the lamp position in the foreground emitting towards the UV exposure volume within the chamber at the rear of the simulation volume. The aerosols move from left to right and accumulate dose according to the radiation flux at their position. The aerosol particle streamlines were observed to proceed directly across the chamber but did converge into the two exit ports for the chambers shortly after leaving the exposure area. The major influence on the aerosol particle streamlines appears to be the friction around the edges of the flow volume since the flow pattern takes a parabolic flow profile (visible in panels B and C of Figure 1) typical of laminar flow conditions. The color change of the aerosol particles in Figure 1 represents the relative survival of virus throughout the exposure.

**Figure 2:**
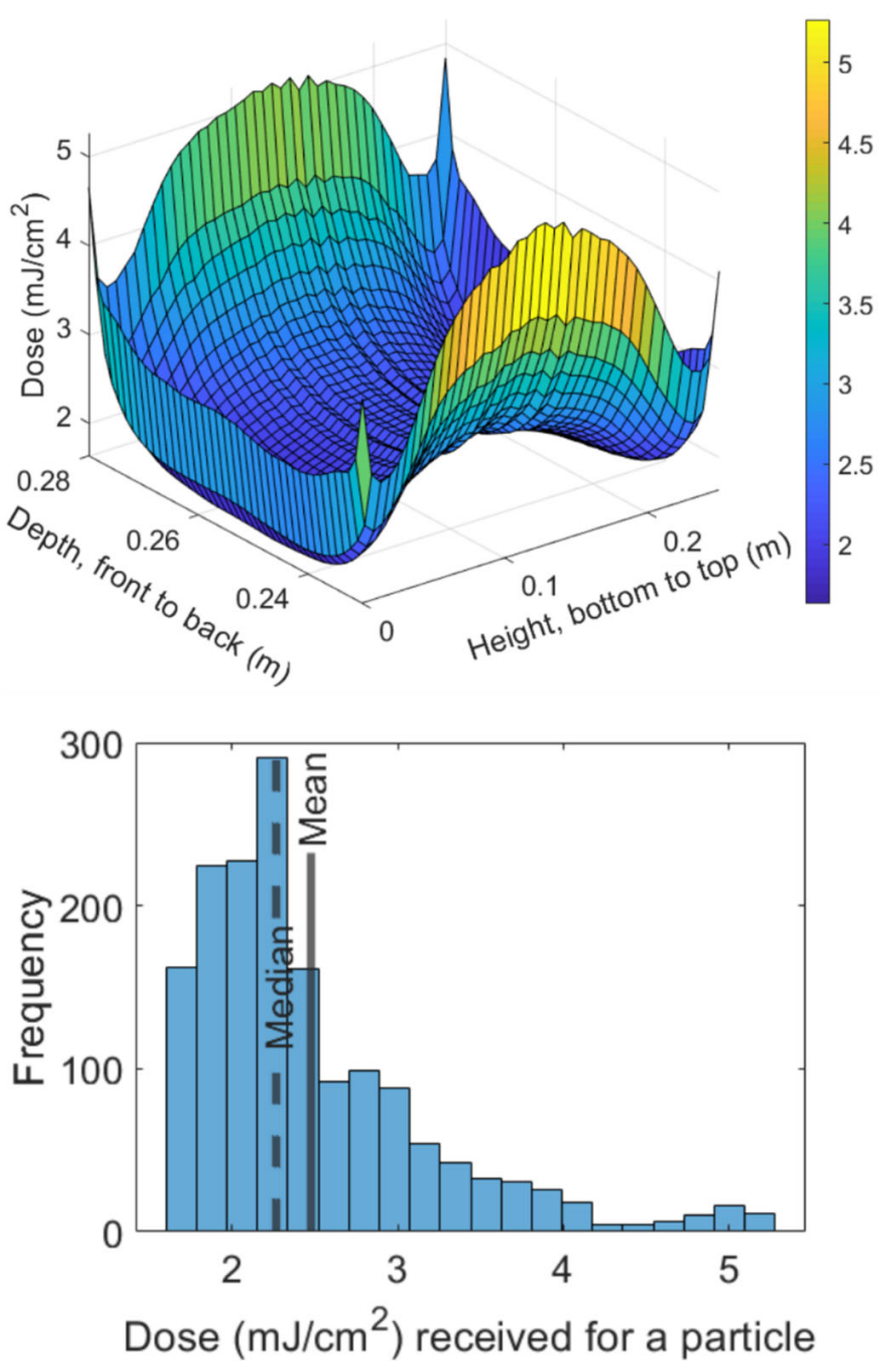
The dose received by the simulated aerosol particles as they travel across the exposure chamber. The surface plot shows the influence of the starting position of the particle on the total dose received. The x-axis on the plot is the depth of the chamber (0.23 m to 0.28 m), which defines the space from the front of the chamber to the back wall of the chamber. The y-axis of the plot is for the height of the chamber. A histogram plot of the frequency of each dose among the 1600 simulated particles indicates that the dose distribution is right skewed.

The total flux upon each simulated aerosol particle was tracked through the chamber, and the cumulative doses are plotted in Figure 2 in both a surface plot and a histogram. Due to the laminar flow conditions, aerosol particles entering at the same position followed the same path though the chamber and hence experienced the same radiation exposure and underwent the same virus inactivation. However, aerosol particles entering through different positions at the inlet took different paths through the chamber, experienced varying levels of radiation exposure, and hence experienced different doses. Consequently, the dose experienced by the virus can be mapped as function of position at the inlet face of the chamber. This is presented in Figure 2 showing a higher dose being received to virus close to the chamber’s edges which is a result of the slowing of the fluids and, therefore, an extension of the dwell times. The mean and median dose received by a particle for the simulation were, respectively, 2.47 ± 0.71 mJ/cm^2^ and 2.26 mJ/cm^2^. The mean dose was used for modeling of the virus susceptibility.

The different doses delivered to the aerosol particles in the experiment were obtained with a combination of precision wire meshes to reduce the total exposure time by blocking a portion of the total far-UVC exposure. The different mesh blocking combinations are listed in Table 1, along with the range of doses ultimately applied to the aerosol.

### 3.2. Viral Inactivation Results

**Figure 3:**
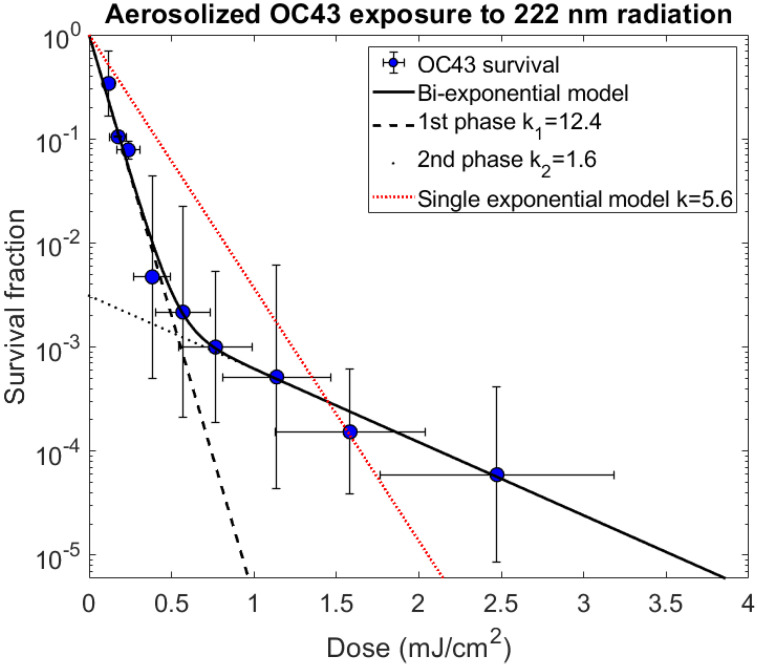
Survival fraction of coronavirus OC43 exposed to 222 nm radiation fitted with the two-phase decay model. The circle markers represent the mean survival values for a given mean exposure dose. The x-error bars show the standard deviation of the doses for the 1600 particles in the simulation, and the y-error bars show the standard deviation of the experimental repeats of survival fraction. The two-phase decay model fit to the data is included on the graph (solid line), as well as lines representing the decay of the first and second stages separately (dashed and dotted lines, respectively). The single exponential model fit to the same data is included for comparison.

As described in the Methods section, the susceptibility of aerosolized HCoV-OC43 to inactivation by 222 nm radiation was analyzed using the standard TCID_50_ (50% Tissue Culture Infectious Dose) assay [41,42] to determine the inactivated fraction. Results for the nine exposure doses are shown in Table 1 and Figure 3.

The earlier published results [14] on the susceptibility of HCoV-OC43 were fit to a single-exponential dose–response model (Equation (2)). However, the wider range of doses utilized here allows us to test whether the single-exponential model provided an adequate fit to the expanded data set, relative to the commonly-used [7] two-phase bi-exponential model (Equation (1)). As described in the Methods, the two models and their fits to the data were compared using the Akaike information criterion, resulting in an extremely large difference (ΔAICc = 47.3); the corresponding very small value of the evidence ratio [*exp*(−47.3/2) = 5 × 10^−11^] indicates that the bi-exponential model provided a significantly better description of the data than the single-exponential model—in agreement with visual inspection of the data in Figure 3.

Based on the two-phase bi-exponential model fits, the first phase, representing the more susceptible fraction of the virus population, had an estimated susceptibility constant of *k*_1_ = 12.4 ± 0.4 cm^2^/mJ and described the behavior of approximately 99.7% (1 − *f*, with *f* = 3.1 × 10^−3^ ± 5.4 × 10^−4^) of the virus population. The second phase of the bi-exponential model, representing the less susceptible fraction of the virus population, was found to have an estimated susceptibility constant of *k*_2_ = 1.6 ± 0.1 cm^2^/mJ and describes the behavior of 0.31% ± 0.054% of the virus population. By contrast, the single exponential fit to the data yielded a susceptibility constant of *k* = 5.6 ± 0.9 cm^2^/mJ. All of the susceptibility constant uncertainty values reported are standard errors.

## 4. Discussion

With far-UVC radiation of increasing interest as a promising technology to limit airborne disease transmission in occupied indoor spaces, it is important to be able characterize the efficacy of this technology. This study utilized a combination of experimental virus inactivation data and state-of-the-art far-UVC dosimetry to more accurately characterize the susceptibility of airborne human coronavirus to 222 nm far-UVC radiation.

Our earlier study [14] on inactivation of airborne HCoV-OC43 using 222 nm far-UVC yielded a single-phase susceptibility constant estimate of 5.9 cm^2^/mJ, similar to what we estimated in the current study (5.6 cm^2^/mJ) using a single-phase single-exponential model. However, the much narrower spread of doses used in the earlier study [14] precluded analysis with a two-phase model. In the current study the results, covering a much wider dose range, clearly showed that a two-phase bi-exponential model provided a much better description of the dose–response data. 

While observations of a biphasic bi-exponential dose response, in effect the existence of a small proportion of UV resistant pathogens, are common in the field of ultraviolet disinfection [7,48], explanations for this effect are still unclear. In some situations, clumping or clustering may provide increased resistance to a subpopulation, since the outer microbes can act as a photo-protective shield [7,49]. Recent work by Kowalski et al. [48] has incorporated Mie scattering to model a biphasic dose response related to self-shielding effects. Alternatively, biphasic dose responses could be caused by a small UV-resistant subpopulation among the target pathogens. Hiatt [50] commented on deviations from the single exponential model and grouped the possible explanations as either vitalistic, where the deviations are due to heterogeneity of the microorganisms, or mechanistic, which attributes deviations to factors during the reactions [50]. However, the overall influence on the observed bi-exponential response from both vitalistic or mechanistic factors remains unclear for both experimental setups and real-world implementations.

Irrespective, however, of the mechanism(s) underlying the radioresistant fraction, based on the data analysis here using the two-phase model, we estimate that 99.7% of the viral population—the more radiosensitive subpopulation—was inactivated with a susceptibility constant of *k*_1_ = 12.4 cm^2^/mJ, more than twice the susceptibility estimate that was derived using the single-exponential model. This increased susceptibility estimate implies that a much lower far-UVC dose will be required than previously thought in order to inactivate the great majority of the airborne aerosolized pathogens in a room environment. The increased susceptibility also suggests that more viruses will be inactivated for a given dose; therefore, installations of far-UVC, which are dose limited by regulatory guidelines, will achieve target inactivation doses, e.g., a 99.9% inactivation dose, in a shorter time while operating with the same exposure conditions.

Further improvements to estimates of far-UVC efficacy against aerosolized virus could be beneficial. Tests and simulations which examine effects on virus susceptibility from variables such as different aerosol sizes or variations in the composition of the suspending media containing the virus would be of interest to real-world disinfection situations. Additions to the model could also improve the accuracy of the current simulation. For example, expanding the model to incorporate additional portions of the experimental chamber prior to the exposure volume could improve the understanding of the expected particle trajectories through the system, and this could ultimately lead to better dose estimation. Furthermore, additional considerations of effects such as reflection and shadowing by the aerosols on radiation transport would provide information on the possible influence of these factors on virus susceptibility.

In summary, these new results provide added support for the suggestion that far-UVC could be a highly efficient modality for reducing the level of airborne pathogens in occupied public spaces. The results suggest that the achievable reduction in airborne pathogens—at the low far-UVC exposures which are consistent with current regulatory limits—will be significantly greater than previously [14,34] estimated.

## Figures and Tables

**Table 1:** Summary of exposure conditions and survival results from the exposure of aerosolized HCoV-OC43 to 222 nm radiation. Combinations of mesh screens, their transmission percentage, and the shielding of half of the exposure window permitted the range of exposure doses used for testing. The mean dose with 100% intensity, achieved with full exposure time and no meshes present, was determined using the computational model. The standard deviation is abbreviated as SD.

Mesh on Lamp % Open Area	Mesh on Chamber % Open Area	Exposure Time (s)	Percentage of Maximum Dose	Mean Dose (mJ/cm^2^)	Survival Fraction	ln(Survival Fraction)	SD ln(Survival Fraction)
None	None	23	100%	2.47	5.94 × 10^−5^	−9.73	1.94
None	64%	23	64%	1.58	1.53 × 10^−4^	−8.78	1.38
None	46%	23	46%	1.14	5.16 × 10^−4^	−7.56	2.46
None	31%	23	31%	0.767	9.99 × 10^−4^	−6.91	1.67
None	46%	11.5	23%	0.569	2.17 × 10^−3^	−6.13	2.34
None	31%	11.5	15.5%	0.383	4.72 × 10^−3^	−5.36	2.24
31%	31%	23	9.61%	0.238	7.82 × 10^−2^	−2.55	0.191
31%	46%	11.5	7.13%	0.176	1.05 × 10^−1^	−2.26	0.0141
31%	31%	11.5	4.80%	0.119	3.40 × 10^−1^	−1.08	0.714

## Data Availability

Data have been included in the text.

## Supplementary Materials

The following supporting information can be downloaded at https://www.mdpi.com/article/10.3390/v14040684/s1. Video S1: far_UVC_aerosol_chamber_simulation.mp4. Figure S1: Normalized spectral irradiance of far-UVC source.
