# Supplementary figures and images for: Inactivation Rates for Airborne Human Coronavirus by Low Doses of 222 nm Far-UVC Radiation
Source: Viruses. 2022 Mar 25;14(4):684. doi: 10.3390/v14040684 (PMC9030991; doi:10.3390/v14040684)

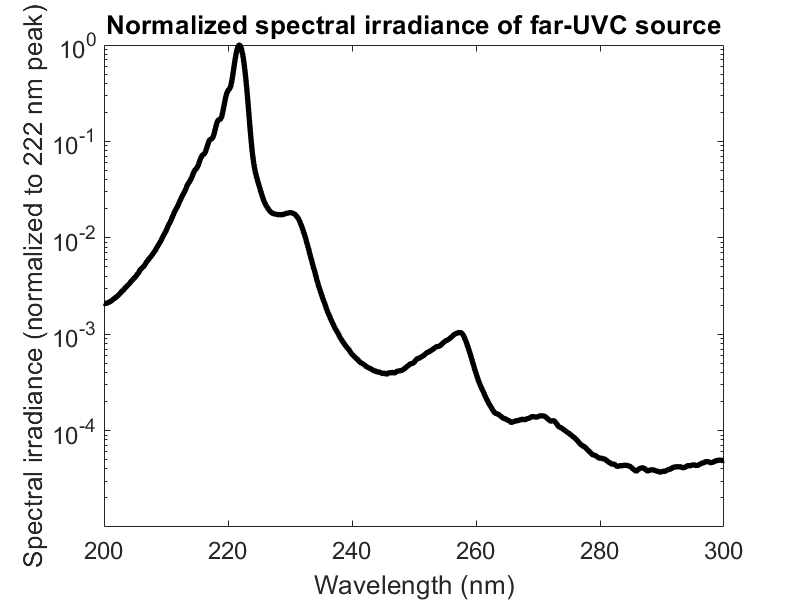

Supplement: Supplementary file 1 [file viruses-14-00684-s001.zip › Figure S1.png]
